# The impact of apoptosis-inducing MAPK and glycolytic pathways modulated by *Aloe vera* and royal jelly in lung and colorectal cancer

**DOI:** 10.1007/s12032-025-02606-7

**Published:** 2025-01-21

**Authors:** Tuğba Kul Köprülü, Bahar Gezer, Burçin Erkal Çam

**Affiliations:** 1 Experimental Medicine Application and Research Center, Validebağ Research Park, University of Health Sciences, Altunizade, Kalfaçeşme Street, Üsküdar, 34662 İstanbul, Turkey; 2 Department of Molecular Medicine, Hamidiye Institute of Health Sciences, University of Health Sciences, 34668 İstanbul, Turkey; 3 Department of Molecular Biology and Genetics, Faculty of Arts and Sciences, Yıldız Technical University, Esenler, İstanbul, Turkey

**Keywords:** Glycolytic capacity, Extracellular acidification rate, Transcriptome profiling, MAPK signaling, *BCL-2*, *BAX*

## Abstract

Lung and colon cancer are among the most commonly diagnosed and fatal cancer types in the world. Due to their metastatic properties, they complicate the treatment process and pose a great threat to human health. These aggressive types of cancer are resistant to chemotherapy drugs. Therefore, it is extremely important to investigate the therapeutic effects of natural compounds. In our previous study, effective doses of Royal Jelly (RJ) (100 mg/mL) and *Aloe vera* (AVE) (20 µg/mL) were determined and tested separately and in combination on lung and colorectal cancer cells. Glycolytic capacities were determined using the Seahorse XFe24 Analyzer, total transcriptome profiles were sequenced using NovaSeq 6000, and *BAX* and *BCL-2* gene levels were determined using RT-qPCR. It was seen that RJ and RJ + AVE affected glycolytic capacity and more genes in lung cancer cells. In HT29, AVE alone was seen to reduce glycolytic capacity and RJ + AVE combination was seen to reduce the expression level of genes related to cell proliferation and cycle. After RJ + AVE treatments, the apoptotic process which is triggered via MAPK pathway was found in lung cancer. Moreover, *BAX* levels increased and *BCL-2* levels decreased both lung and colorectal cancer cells. It was observed that the combination of RJ and AVE affected the glycolysis process, cell cycle, proliferation and apoptosis on lung and colorectal cancer. In particular, the combination of RJ + AVE was found to be more effective on lung cancer.

**Supplementary Information:**

The online version contains supplementary material available at 10.1007/s12032-025-02606-7.

## Introduction

Lung cancer is a disease that causes 1.6 million deaths worldwide and 2.20 million people are diagnosed every year [1, 2]. Non-small cell lung cancer (NSCLC), which has tumorigenic properties, accounts for the majority of this condition. This high-incidence disease has a high lethality rate for the human population [3]. Colorectal cancer (CRC) is a type of cancer that is common worldwide and has somatic and genetic mutations [4]. Colonic epithelial cells evolve into invasive and malignant stages over time. Cells that become metastatic begin to differ biochemically and genetically. It is the fourth deadliest type of cancer globally [5]. The metabolism of cancer cells differs from normal cells. This impaired metabolic activity causes cancer cells to grow and multiply pathologically rapidly, and the cells acquire malignant properties and form tumors [6]. Impaired metabolic pathways inhibit the tumor-suppressor genes of malignant cells, causing the cell to proliferate unlimitedly and escape from apoptotic pathways. It is necessary to reprogram its metabolic pathways in order to provide the necessary energy to these cancer cells that have grown indefinitely [7]. While in normal cells, pyruvate is broken down for energy production, in cancer cells, glycolysis occurs independently of oxygen, and lactate production begins as pyruvate inhibition occurs in the mitochondria. The fact that cancer cells prefer glycolysis even in the presence of oxygen is called the Warburg effect [8, 9]. In NSCLC, lactate accumulates due to aerobic glycolysis due to the malfunction of some metabolic pathways and genes [10]. Cells use the energy from glycolysis to prevent tumor inhibition and increase immune suppression [11]. Likewise, in CRC, aerobic glycolysis causes cell proliferation, growth, and metastasis [12]. According to the studies made for various types of cancer, it is mentioned that the most important proteins involved in the glycolytic pathway are Hexokinase (HK), Pyruvate kinase (PK), and glucose transporters (GLUT) proteins. While HK and PK proteins are involved in the conversion of glucose to pyruvate, GLUT proteins transport glucose to cell. It has been found that by inhibited or dysfunctionated HK proteins involved in the mechanism of glycolysis, glucose metabolism is inhibited in cancerous cells and apoptosis is induced [13–15]. In addition, thanks to the inhibition of GLUT proteins, cancerous cells enter apoptosis [14]. These studies show that the glycolytic pathway will be used as an alternative treatment target for cancerous cells [13–15]. Oncogenes that convert aerobic respiration into glycolysis cause the synthesis of macromolecules and rapid ATP production in this type of cancer [16]. These oncogenes activate transcription factors that initiate the glycolytic pathway [17] and regulate cancer genetic metabolism, avoiding oxidative phosphorylation used by normal cells [18]. Extracellular acidification is used to measure the lactate production of cells. This ratio can be found by looking at the respiration rate of the cells, the CO_2_ level produced and the pH environment [19]. In the absence of oxygen and nutrients, cancer cells increase lactate production and release it, causing the balance in the extracellular matrix (ECM) to be disrupted and the pH level inside and outside the cell to change [20]. Another pathway involved in cell growth, differentiation, migration, and apoptosis in cancerous cells is the mitogen-activated protein kinase (MAPK) pathway. This pathway is activated or inhibited under control in normal cells. Uncontrolled growth, development, and metastasis are observed in cancer cells because of that there is no control of this pathway. In addition, the MAPK pathway is also involved in the control of the apoptosis mechanism. Due to the disregulation of MAPK pathway, cancer cells become resistant to apoptosis. If this pathway can be controlled, the growth of cancerous cells can be prevented, and at the same time the cells can be dragged into apoptosis [21]. RJ is a natural substance that contains various chemokines and growth factors that have antiinflammatory and anticancer properties. Providing protection against the side effects of drugs used in cancer treatments, RJ also provides cell survival and treatment in a molecular sense [22]. Its major role in the apoptotic process has also been revealed by stimulating tumor-suppressor genes and caspase activity and even suppressing antiapoptotic oncogenes [23]. AVE is a natural product that is rich in vitamins, minerals, and enzymes, has anticancer, antidiabetic, and antiproliferative properties and has a therapeutic effect on the skin [24, 25]. AVE, which contains immunostimulating polysaccharides and bioactive compounds, is used in many cancer treatments [26]. It is a substance that induces apoptosis, especially in lung cancer cells, and also reduces the side effects of chemotherapeutic drugs [27]. AVE also stimulates apoptosis in colon cancer cells via the mitochondrial pathway, and AVE polysaccharides regulate mitochondria metabolism [28]. In our previous study [29], the effective doses of RJ and AVE on lung and colon cancer cells were determined by xCELLigence (RTCA). The main aim of this study was to determine how RJ and AVE alter the glycolytic function, apoptotic process and total transcriptome profile of lung and colon cancer types. In particular, it was to observe whether the combined use of the substances increases the effect they have alone. The reason for the combined use of RJ and AVE is that RJ, a natural substance, protects against AVE-induced necrotic cell death and reduces the side effects of AVE. It is also predicted that the combination will increase the anticancer activity by showing more antiproliferative effect and induce early apoptosis.

## Materials and methods

### Cell culture and preparing of compounds

A549 (CCL-185™, human non-small cell lung cancer) and HT29 (HTB-38™, human colorectal adenocarcinoma) cells were cultured in DMEM (4.5 g/L) (Capricorn Scientific, Germany) medium containing 2% Penicillin/Streptomycin (Capricorn Scientific, Germany) and 10% fetal bovine serum (Capricorn Scientific, Germany). The cells were incubated in cell culture flasks at 37 °C and 5% CO_2_ until they were 80% confluent. Cells detached from the flasks using trypsin/EDTA (Gibco New Zealand) were stained with trypan blue (BioRad, USA) at a ratio of 1:1 and counted on TC20 slides in the BioRad TC20 Cell Counter device. RJ was obtained from Macahel (Artvin, Turkey) and prepared with PBS at a concentration of 1 g/mL. It was stored at 4 °C and in a dark environment. The leaves of AVE were cleaned with distilled water and dried. Gel was obtained from dried leaves and homogenized. The gel was diluted after being centrifuged at 300 g for 3 min and incubated with mixing at 60 °C for 2 h. Finally, centrifugation was performed again and the resulting supernatant was concentrated (3.01 g).

### Seahorse glycolysis stress assay

Extracellular Acidification Rate (ECAR) (mpH/min) of the cells was measured with the Agilent Seahorse XFe24 Analyzer using the glycolysis stress test kit (Agilent Technologies). For device calibration, A549 and HT29 cells were planted on cell culture plates in DMEM (Capricorn Scientific, Germany) media at a concentration of 30,000 cells/well. After the cells were planted, RJ and AVE were added to the cells. All substances were added according to the effective doses determined in our previous study [29]. In the RJ group, RJ was added to the cells at a concentration of 100 mg/mL, and in the AVE group, 20 µg/mL AVE was added to the cells. The procedure was completed by giving 100 mg/mL RJ and 20 µg/mL AVE combination to RJ + AVE group. Culture plates were incubated for 24 h at 37 °C and 5% CO_2_. Then, 10 mM glucose, 1 µM oligomycin, and 50 mM 2-deoxyglucose (2-DG) was added to sensor plates. These plates were placed on the Agilent Seahorse XFe24 device and scanned. Incubated cell culture plates were placed in the device and run. When the run was finished, analyzes were performed using Seahorse Wave Controller Software 2.4.

### Total RNA library preparation and sequencing

Total RNA libraries were constructed to generate transcriptome profiles of RJ and AVE, the effective dose of which was determined in the previous study [29]. 100 mg/mL RJ and 20 µg/mL AVE and the combination of two doses of the substance were applied to A549 and HT29 cell lines. RNA of the cells was isolated 24 h after substance application (Bio Basic, Canada). Quantification and qualification of isolated RNAs were measured with Qubit 4.0 Fluorometer and TapeStation 4150 (Agilent, USA). Measurements were obtained at concentrations of 250–1000 ng and RIN values in the range of 7.7–9.8. Stranded total RNA sequencing library was performed with the QIAseq FastSelect™ RNA (Qiagen, Germany) library kit. It was entered into the library with an amount of 100 ng of RNA. The library was constructed step by step according to the protocol provided by the manufacturer. First, cDNA was obtained without RNA-specific heat degradation by rRNA removal reaction. The samples were amplified with the 10 base pair indexes from the kit. The libraries attached to the beads went through washing steps and libraries with a value of approximately 50 nM were obtained. A 2 nM pool of all samples was assembled and the pool was sequenced on the NovaSeq 6000 (Illumina, USA). Bioinformatics analyses were performed with the GeneGlobe (Qiagen, Germany) system.

### Enrichment analysis of DEGs

In the DEGs (Differentially Expressed Genes) determined for each group, those with Bonferroni values less than 0.05 and logFC values greater than 1 and less than −1 were filtered. Pathway (shinyGO 8.0-KEGG pathway), gene ontology, and PPI analyses (g:Profiler-BP;MF;CC) of hub genes with neighbors were performed separately. PPI analyses and hub gene analyses for each group were performed with the help of Cytoscape tool with Cytohub program. As a result of all these analyses, p/FDR values less than 0.05 were considered significant.

### Quantitative real-time PCR (RT-qPCR)

RJ and AVE, whose effective doses were determined in the previous study [29], were applied to A549 and HT29 cells and the levels of genes involved in the apoptotic process in the cells were determined by real-time PCR. 100 mg/mL RJ, 20 µg/mL AVE, and the combination of both doses were given separately to A549 and HT29 cells, and 24 h later, the total RNA of the cells was isolated using the RNA extraction kit (Bio Basic, Canada) according to the protocol provided by the manufacturer. The concentration of the obtained RNAs was measured with a Qubit 4.0 Fluorometer and converted into 2 ng cDNA with the RT^2^ First Strand Kit (Qiagen, Germany). RT-qPCR was performed on a Roche Light Cycler 480 II using the RT^2^ SYBR Green FAST Master Mix kit (Qiagen, Germany). Each sample was studied in triplicate. *GAPDH* primer (Qiagen, Germany) was used as the reference gene, and relative gene expressions were examined using *BCL-2* and *BAX* primers (Qiagen, Germany). The primers used are as in Table 1. Statistical results were calculated with GeneGlobe (Qiagen, Germany).

**Table 1:** Primer sequences of *GAPDH*, *BAX,* and *BCL-2* genes

Gene	Accession no	Forward sequence	Reverse sequence	*T*_M_
*GAPDH*	NM_002046.5	GTCTCCTCTGACTTCAACAGCG	ACCACCCTGTTGCTGTAGCCAA	60 °C
*BAX*	NM_004324.3	TCTGACGGCAACTTCAACTG	TTGAGGAGTCTCCCCAACC	60 °C
*BCL-2*	NM_000633.2	ATCGCCCTGTGGATGACTGAGT	GCCAGGAGAAATCAAACAGAGGC	60 °C

### Statistical analysis

GraphPad Prism Version 10.2.3 program was used for RT-qPCR analyses. Two-way ANOVA analysis was followed by Tukey's multiple comparisons test for statistical comparison between all groups.

## Results

### Analysis of ECAR in A549 cells

**Fig. 1:**
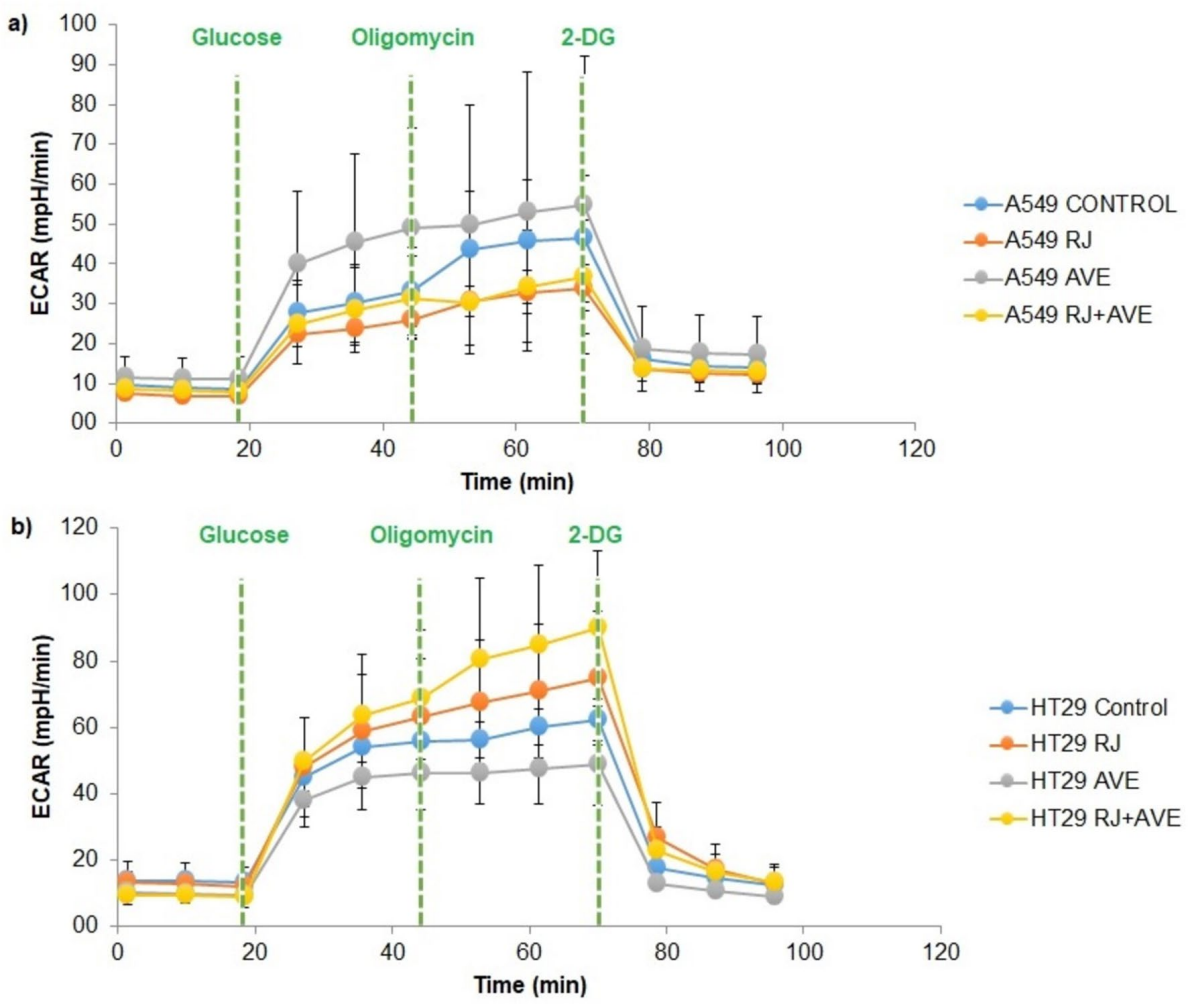
**a** ECAR (mpH/min) levels of A549 cells treated with RJ and AVE, **b** ECAR (mpH/min) levels of HT29 cells treated with RJ and AVE. *CONTROL* cells without treatment, *RJ* cell treated with 100 mg/mL RJ, *AVE* cells treated with 20 µg/mL AVE, *RJ + AVE* cells treated with 100 mg/mL RJ and 20 µg/mL AVE

After basal measurements of A549 cancer cells were taken for the first 20 min, glucose was added to the medium (Fig. 1a). With the administration of glucose, all groups entered the glycolytic pathway and an increase in ECAR (mpH/min) level was observed. Then, with the introduction of oligomycin, mitochondrial ATP production stopped and therefore cancer cells shifted to glycolysis for energy production. For this reason, there was an increase in the ECAR (mpH/min) level again. Finally, 2-DG added to the medium entered into competitive binding with hexokinase and glycolysis was stopped. When the glycolytic capacity is compared between groups, it is seen that the dose that minimizes A549's entry into the glycolysis pathway is 100 mg/mL RJ. At the same time, it seems that the effect of the combined use of 100 mg/mL RJ and 20 µg/mL AVE is very close to the effect of the RJ dose alone. Interestingly, a dose of 20 µg/mL AVE alone triggered a greater preference of A549 for the glycolysis pathway compared to the untreated control group. The fact that RJ alone and in combination with AVE significantly reduces the non-stop glycolysis capacity of A549 allows lactate production to decrease and thus reduce or eliminate mitochondrial respiratory defects in A549.

### Analysis of ECAR in HT29 cells

When Fig. 1b is examined, 20 µg/mL AVE minimized the glycolytic capacity in HT29 cells compared to untreated cells and was more effective than RJ alone and RJ + AVE combination treatments. It is observed that the capacity of the AVE group decreases rapidly after glucose is introduced into the environment. Because in HT29, which is an aggressive cancer cell, while glycolysis continues uninterruptedly, AVE suppresses it and causes a serious decrease, especially in ECAR (mpH/min) value, compared to the control group. When other groups were compared, it was seen that 100 mg/mL RJ alone reduced glycolytic capacity more than the combination of 100 mg/mL RJ and 20 µg/mL AVE, but interestingly, both of these doses put the cells into glycolysis more than the control group.

RJ and AVE substances affected glycolytic capacity in A549 and HT29 cells at different angles. AVE decreased the glycolytic capacity of HT29 cells compared to untreated cells, while AVE increased glycolytic capacity in A549 cells. However, RJ decreased glycolytic capacity in A549 cells and also decreased the glycolytic capacity increased by AVE, bringing it to the same level as its effect alone. In HT29 cells, RJ alone and in combination with AVE did not have as much effect on glycolytic capacity as AVE, that is, it did not decrease glycolytic function like AVE treatment, on the contrary, it increased the glycolytic capacity.

### RNA-Seq analysis

#### Functional enrichment analyses of A549 and HT29 cells treated with RJ and AVE

**Fig. 2:**
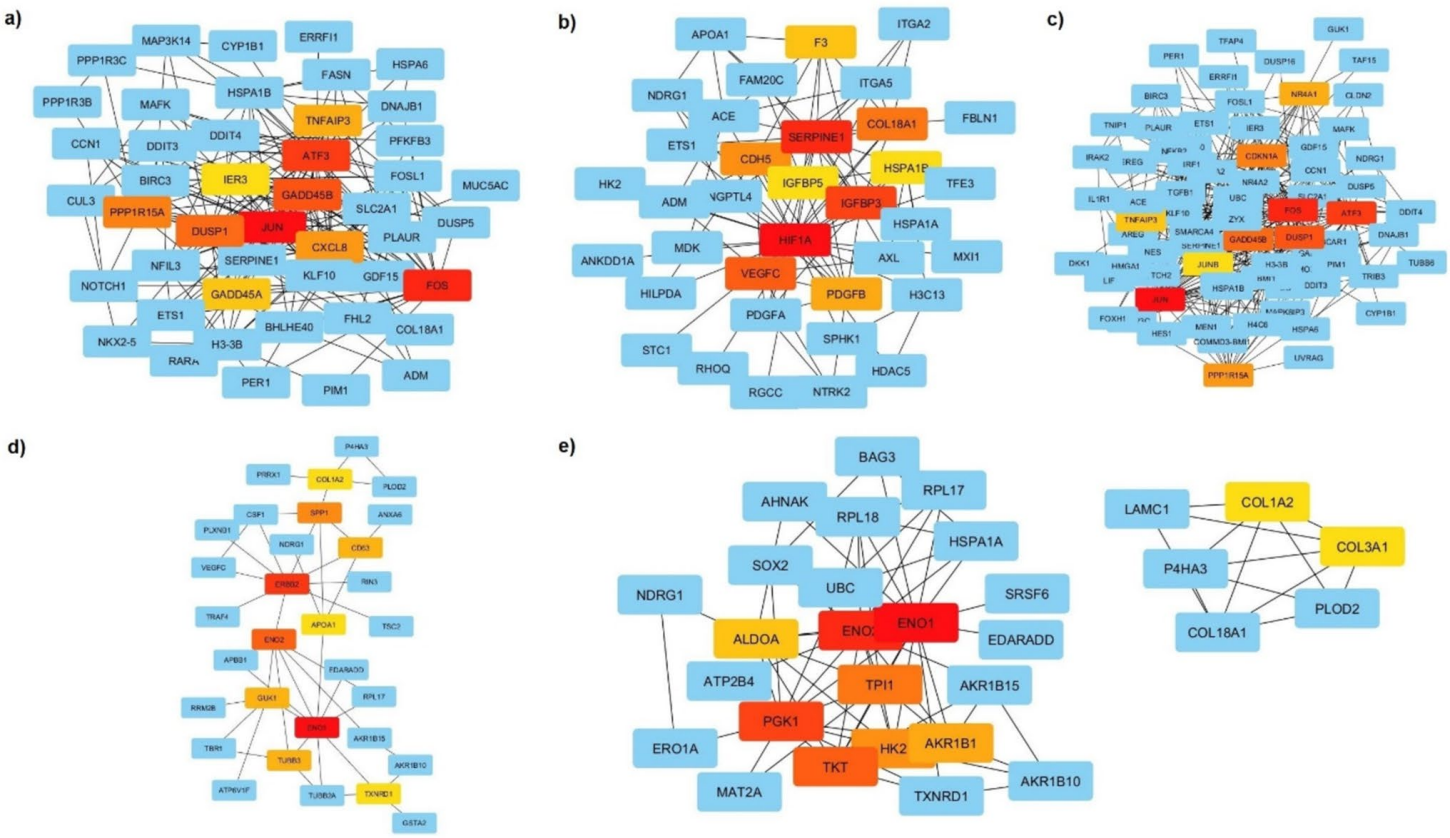
Hub genes network of A549 and HT29 cell lines treated with RJ and AVE. **a** A549 cells treated with 20 µg/mL AVE against non-treated control group, **b** A549 cells treated with 100 mg/mL RJ against non-treated control group, **c** A549 cells treated with 100 mg/mL RJ and 20 µg/mL AVE against non-treated control group, **d** HT29 cells treated with 100 mg/mL RJ against non-treated control group, **e** HT29 cells treated with 100 mg/mL RJ and 20 µg/mL AVE against non-treated control group

These studies revealed that much more genes were impacted by the treatments administered in A549 compared to HT29. The number of DEGs for AA vs. AC:127, AR vs. AC:184, ARA vs. AC:227 (AA vs AC: A549 cells treated with 20 µg/mL AVE against untreated group, AR ve AC: A549 cells treated with 100 mg/mL RJ against untreated group, ARA vs AC: A549 cells treated with 100 mg/mL RJ and 20 µg/mL AVE against untreated group). The number of DEGs for HA vs. HC:54, HR vs. HC:132, HRA vs. HC:108 (HA vs HC: HT29 cells treated with 20 µg/mL AVE against untreated group, HR ve HC: HT29 cells treated with 100 mg/mL RJ against untreated group, HRA vs HC: HT29 cells treated with 100 mg/mL RJ and 20 µg/mL AVE against untreated group) (Supplementary 1). For each group, independent pathway and gene ontology analyses of hub genes with neighbors were carried out. For all groups, PPI analyses of hub genes with neighbors were shown in Fig. 2.

**Fig. 3:**
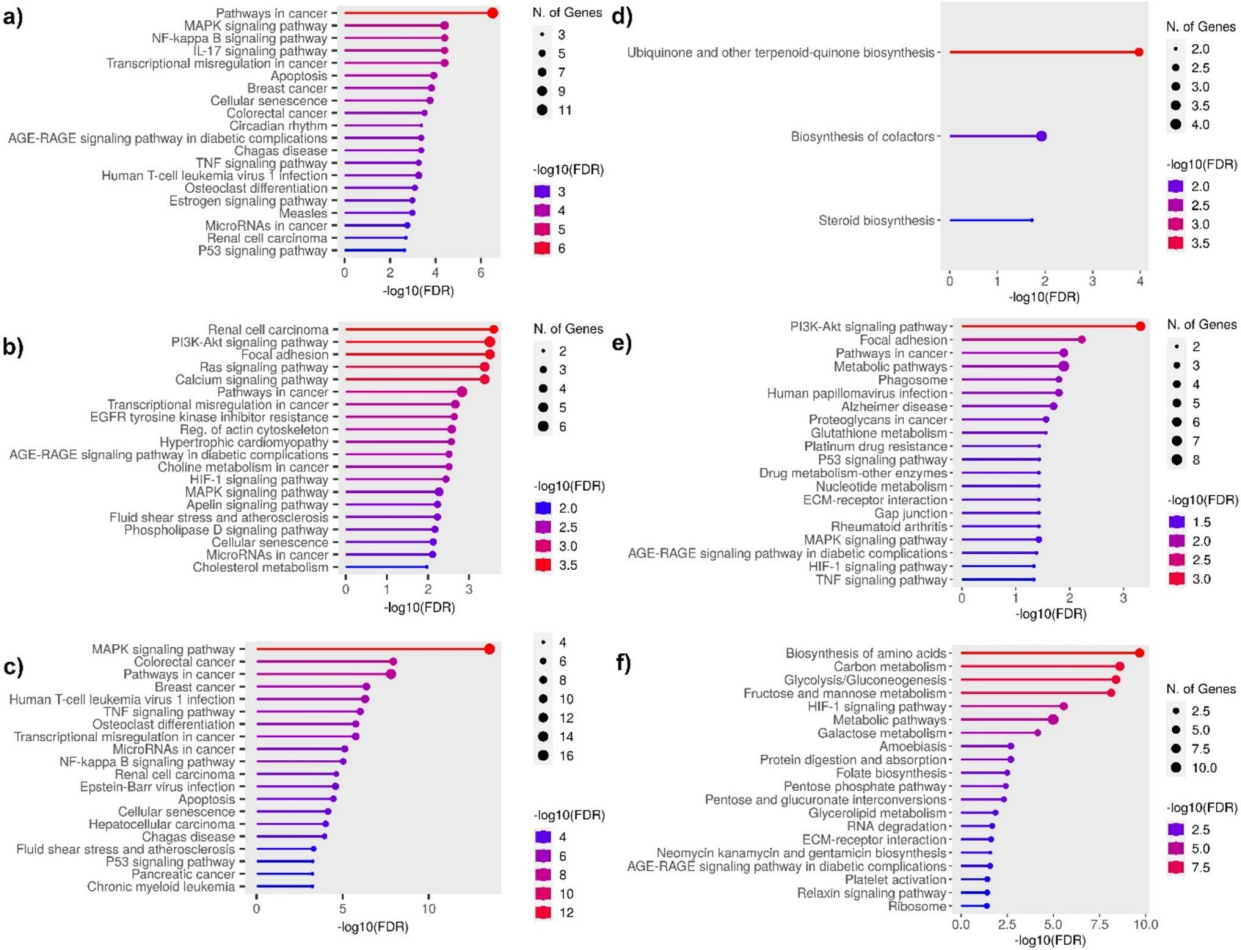
KEGG pathway analyses of A549 and HT29 cell lines treated with RJ and AVE. **a** A549 cells treated with 20 µg/mL AVE against non-treated control group, **b** A549 cells treated with 100 mg/mL RJ against non-treated control group, **c** A549 cells treated with 100 mg/mL RJ and 20 µg/mL AVE against non-treated control group, **d** HT29 cells treated with 20 µg/mL AVE against non-treated control group, **e** HT29 cells treated with 100 mg/mL RJ against non-treated control group, **f** HT29 cells treated with 100 mg/mL RJ and 20 µg/mL AVE against non-treated control group

Pathway analyses conducted in AVE-treated A549 and HT29 cell lines revealed that A549 exhibited significantly more cancer-related pathways than HT29. Pathway in cancer, mitogen-activated protein kinase (MAPK), nuclear factor kappa B (NF-κB), IL-17 signaling pathways, and apoptosis are a few of these pathways (Fig. 3a). On the other hand, HT29 involves cofactor, steroid biosynthesis, and ubiquinone and other terpenoid-quinones (Fig. 3d).

Comparable pathways were discovered to be more prominent in both A549 and HT29 when the RJ treatment was solely used. The impact of RJ on both cells was discovered to be associated with focal adhesion, PI3K-Akt, and pathway in cancer (Fig. 3b,e).

Furthermore, while MAPK, colorectal cancer, pathway in cancer, TNF signaling pathways were found to be prominent pathways in A549 (Fig. 3c), biosynthesis of aminoacid, carbon metabolism, glycolysis/gluconeogenesis pathways were found to be prominent pathways in HT29 with the synergetic effect of RJ and AVE applied together in both cell lines (Fig. 3f).

**Fig. 4:**
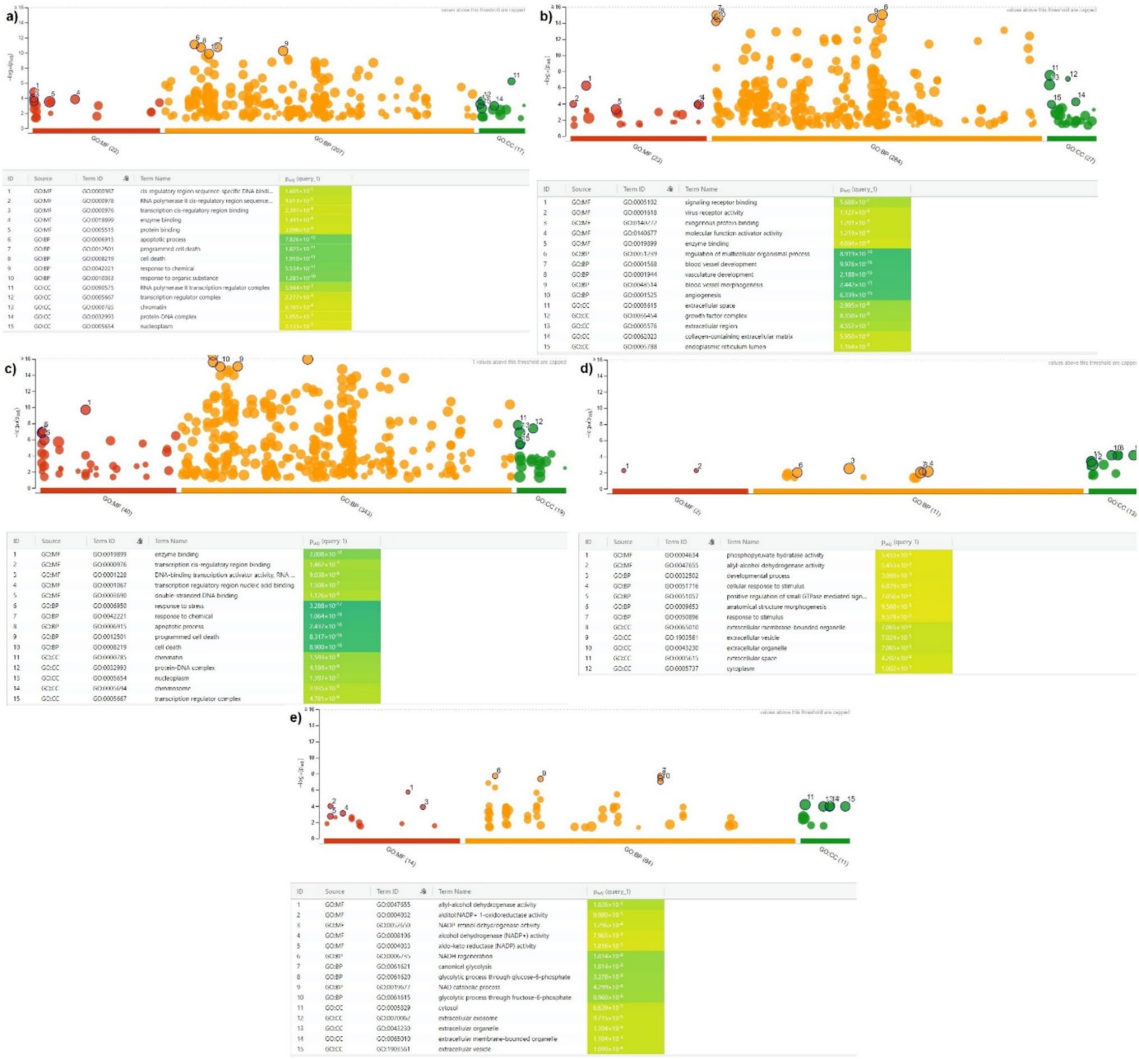
Ontology analyses of A549 and HT29 cell lines treated with RJ and AVE. **a** A549 cells treated with 20 µg/mL AVE against non-treated control group, **b** A549 cells treated with 100 mg/mL RJ against non-treated control group, **c** A549 cells treated with 100 mg/mL RJ and 20 µg/mL AVE against non-treated control group, **d** HT29 cells treated with 100 mg/mL RJ against non-treated control group, **e** HT29 cells treated with 100 mg/mL RJ and 20 µg/mL AVE against non-treated control group

According to gene ontology biological process assessments, genes implicated in programmed cell death are affected by AVE given to the A549 cell line. It is known that genes that control expression through binding to target regions—such as cis-regulatory region DNA binding, enzyme binding, and protein binding—are impacted when the molecular functions are assessed (Fig. 4a). The lack of DEGs that were found to be important made it impossible to identify the molecular mechanisms that collaborate and emerge in the HT29 cell line.

Upon applying just RJ to both cell lines, an assessment of the biological processes in A549 revealed that the genes responsible for blood vessel formation and vasculitis emerged. Mechanisms relating to the binding of signaling receptors and the functioning of viral receptors have been highlighted in molecular function analysis (Fig. 4b). Cellular response to stimulation and the developmental process are two mechanisms involved in the biological process in the HT29 cell line. Phosphopyruvate hydratase and allyl-alcohol dehydrogenase activities were shown to be more significant in molecular function analysis (Fig. 4d).

Finally, response to stress, stimulation, and apoptotic processes were significant in biological process analysis in A549. In contrast, mechanisms including NADH regeneration and the glycolytic process were discovered in HT29 when the synergistic effect of both treatments was assessed in both cell lines. According to molecular function studies, HT29 showed greater significance in NAP-related enzyme activations and allyl-alcohol dehydrogenase activity, whereas A549 showed greater significance in transcription cis-regulatory region binding and enzyme binding (Fig. 4c, e).

**Fig. 5:**
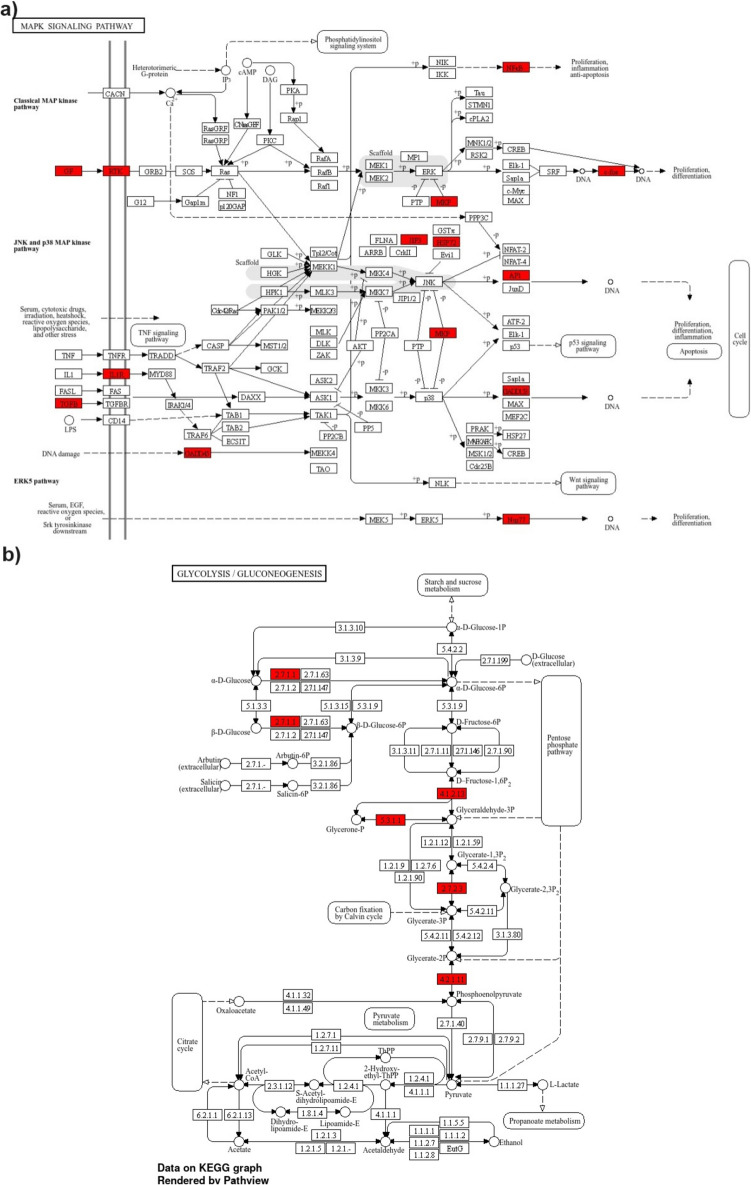
**a** Analyses of hub genes with neighbor related pathway of A549 cells treated with 100 mg/mL RJ and 20 µg/mL AVE against non-treated control group, **b** Analyses of hub genes with neighbor related pathway of HT29 cells treated with 100 mg/mL RJ and 20 µg/mL AVE against non-treated control group

Upon evaluating the combination of RJ and AVE, it was shown that MAPK (FDR: 3.0E-14; 18/294 genes) was an important pathway in hub genes with neighbors in A549. *JUN*, *FOS*, *AREG*, *EREG*, *NFKβ2*, *DUSP1*, *DUSP5*, *HSPA6*, *MAPK8IP3*, *DDIT3*, *HSPA1B*, *EPHA2*, *GADD45B*, *GADD45A*, *TGFβ1,* and *IL1R1* are some of the genes that contribute to making up this group. All gene expression was found to be elevated except for *IL1R1*. In Fig. 5a, genes related to the MAPK pathway are highlighted.

The combination of RJ and AVE brought to light the biosynthesis of amino acid (FDR: 2.8E-10; 7/74 genes) and glycolysis and gluconeogenesis pathway (FDR: 1.6E-08; 6/67) when the same investigations were conducted for HT29. *MAT2A* which is found in biosynthesis of amino acid pathway was shown to be down-regulated among the identified genes, whereas *TPI1*, *PGK1*, *TKT*, *ENO2*, *ENO1*, and *ALDOA* which are found in glycolysis and gluconeogenesis pathway showed elevated expression (Fig. 5b).

#### *BAX* and *BCL-2* expression in A549 ve HT29 treated with RJ and AVE

**Fig. 6:**
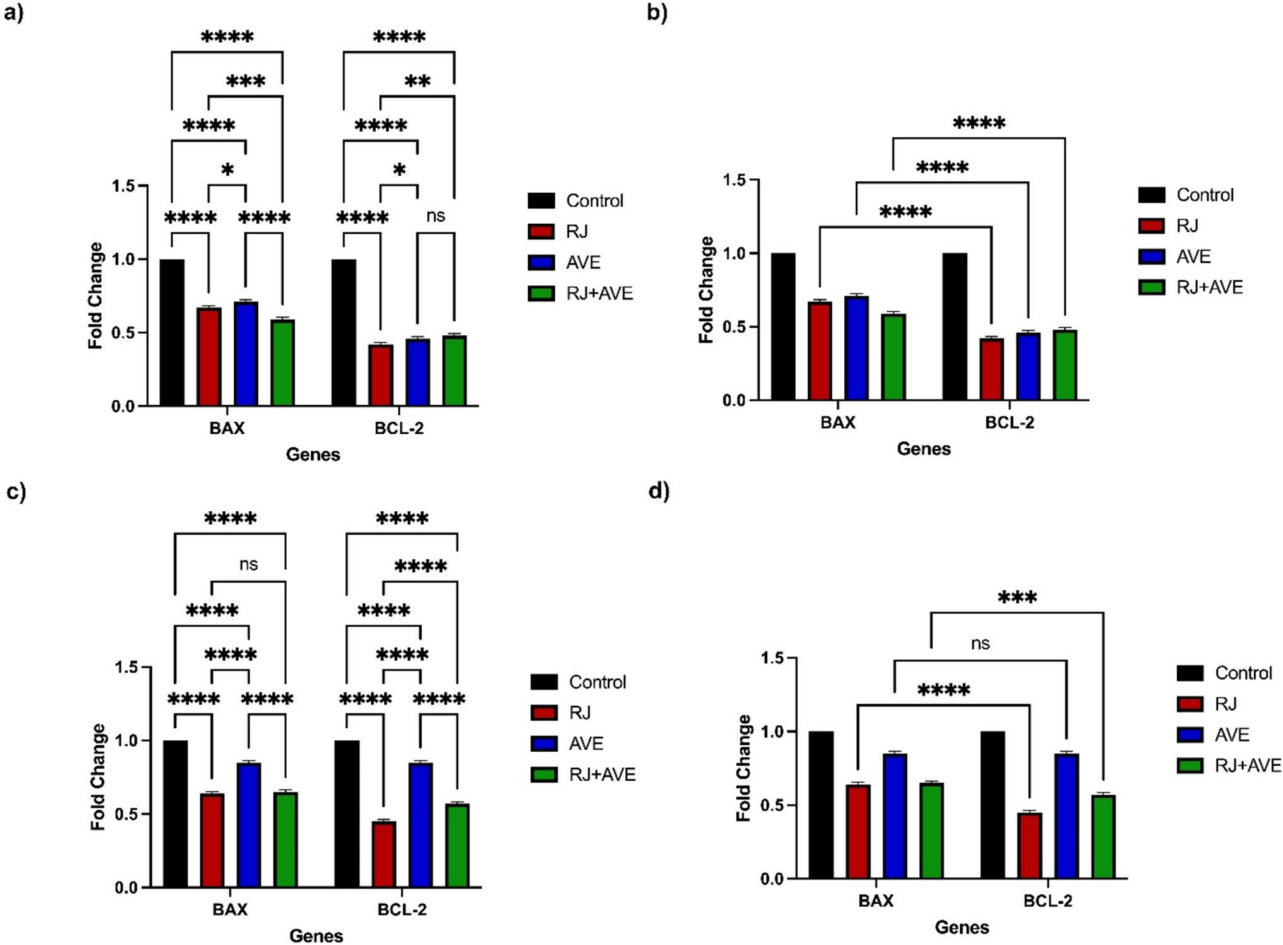
*BAX* and *BCL-2* expression of A549 and HT29 cells treated with RJ and AVE. **a** Fold change in gene expression of A549 cells comparison of each group, **b** fold change in gene expression of A549 cells comparison of *BAX* and *BCL-2* levels, **c** fold change in gene expression of HT29 cells comparison of each group, **d** fold change in gene expression of HT29 cells comparison of each group. *Control* untreated group, *RJ* 100 mg/mL RJ treatment, *AVE* 20 µg/mL AVE treatment, *RJ + AVE* 100 mg/mL RJ treatment and 20 µg/mL AVE treatment (*p < 0.05, **p < 0.01, ***p < 0.001, ****p < 0.0001, *ns* non-significant)

*BAX* and *BCL-2* are known to be genes involved in the apoptotic process. When Fig. 6a is examined, it is seen that the pro-apoptotic *BAX* levels of AVE-treated (*p* < 0.0001) cells in A549 cells are higher than the *BAX* levels of RJ- and RJ + AVE-treated cells. The antiapoptotic *BCL-2* levels of RJ-treated cells (*p* < 0.0001) are significantly reduced compared to the control group. When the *BAX* and *BCL-2* (*p* < 0.0001) groups are examined among themselves (Fig. 6b), it is seen that *BAX* levels are significantly higher than *BCL-2* levels. A similar situation is observed in HT29 cells. There is a significant increase in the *BAX* levels of AVE-treated cells. *BCL-2* levels in RJ-treated (*p* < 0.0001) cells are very low compared to the control group (Fig. 6c). When the *BAX* and *BCL-2* groups are compared with each other, it is observed that the *BCL-2* levels in the RJ-(*p* < 0.0001) and RJ + AVE-treated (*p* < 0.001) groups are lower than the *BAX* levels, but there is no significant difference between the AVE-treated groups (*p* > 0.9999) (Fig. 6d).

## Discussion

Lung cancer is one of the most diagnosed and leading cancer types in the world. Colon cancer follows this list closely. Mutated pathways in cancer metabolism cause cancer cells to constantly grow, proliferate, and spread. These aggressive types of cancer show high resistance to chemotherapy drugs, and these drugs cause side effects in patients over time. Recently, natural products have been tested on cancer types [30, 31]. The main goal of this study is to determine how RJ and AVE change the glycolytic function, apoptotic process, and total transcriptome profile of lung and colon cancer types. In particular, it is to observe whether the use of substances together increases the effect they have on their own. When the results obtained from the Seahorse XFe24 analyzer are examined (Fig. 1a, b), it is seen that A549 cells treated with RJ and RJ + AVE reduce the ECAR (mpH/min) level compared to untreated A549 cells. This suppresses uncontrolled proliferation by reducing the desire of cancer cells to consume ATP. This situation is different in HT29 cells. When Fig. 1b is examined, the use of only AVE decreased the ECAR (mpH/min) level compared to control cells and reduced glycolytic capacity. Based on this, it can be said that the conversion of glucose to lactate decreased due to the natural compounds that cause difficulties in the uptake of glucose into the cell, or inhibition of HK and PK which are involved in the glycolysis pathway. As mentioned in the introduction section, it can be said that these reasons caused the cell to enter apoptosis.

Upon analysis of the transcription profile, it was shown that a greater number of genes were expressed differently in A549 compared to HT29, particularly when both medications were taken individually or in combination. When the synergetic effect of both treatments was evaluated, it was found that MAPK and TNF signaling pathways were particularly prominent in the A549 cell line, while biosynthesis of amino acids, carbon metabolism, glycolysis/gluconeogenesis mechanisms were more significant in HT29.

Enzymes known as MAPKs are members of the extensive family of serine/threonine protein kinases. In addition to being engaged in regulating many other cellular processes (including differentiation and survival). MAPK plays a significant role in channeling proliferative activity from the cell cytoplasm to the nucleus. Transmitting signals from external stimuli, such as hormones, growth factors, cytokines, and intracellular important chemicals, is how this signaling process got started. In NSCLC, aberrant MAPK expression is a rather common occurrence. Cell differentiation, proliferation, drug resistance, and apoptosis are all modulated by the MAPK pathway. There is mounting evidence linking deregulated MAPK signaling to a variety of malignant cancers, including non-small cell lung cancer [32–34].

Growth arrest and DNA damage-inducible 45 alpha and beta (*GADD45A*, *GADD45B*) genes, which are involved in the MAPK pathway and overexpressed in our study, have important roles especially in cancer mechanism. In the lung cancer study conducted by DO et al., [35] they showed that *GADD45B* decreased with increased expression of the *TFAP2C* which is a transcription factor. This decrease supported cell proliferation and cell motility in NSCLC cells. Therefore, it has been suggested that this gene may act as a tumor-suppressor gene in NSCLC cells and may be targeted for therapeutic purposes. Carfilzomib used in a study by Yang et al. [36] inhibited the growth of lung adenocarcinoma by increasing the expression of the *GADD45A* gene both transcriptional and protein level. It is also reported that *GADD45A* increases in cancer cells after the application of many anticancer treatments and helps to increase the effectiveness of the treatments used [37–39].

Depending on the type of cancer, emodin—which is also present in several plants, such as AVE—has both favorable and unfavorable effects on the apoptotic process. When it was administered to the A549 cell line, it was shown to cause cytochrome c release, activation of caspase-2, −3, and −9, and apoptosis in a study by Su et al. [40]. Reactive oxygen species (ROS), disruption of the mitochondrial membrane potential (Dcm), inactivation of ERK and AKT, a decrease in mitochondrial *BCL-2* content, and an increase in mitochondrial *BAX* content were all associated with these events.

Shen et al. [41] have demonstrated in a different study that aloe-emodin triggered autophagy in A549 cells by activating MAPK signaling, inhibiting the Akt/mTOR pathway, and causing caspase-dependent apoptosis.

A study reported that 10-HDA, royal jelly acid, inhibited the proliferation of three types of human lung cancer cells and had no significant toxic effect on normal cells. Accompanied by reactive oxygen species, 10-HDA induced A549 cells to apoptosis by regulating mitochondrial-associated apoptosis and caused cell cycle arrest in the G0/G1 phase in a time-dependent manner. Meanwhile, 10-HDA was also reported to regulate MAPK, signal transducer and activator of transcription 3 (STAT3), and NF-κB signaling pathways by increasing the expression levels of phosphorylated c-Jun N-terminal kinase, p-p38 and I-κB and additionally decreasing the expression levels of phosphorylated extracellular signal-regulated kinase, p-STAT3, and NF-κB [42].

After considering all of these findings, it is believed that RJ and AVE in particular cause apoptosis, which is achieved via phosphorylating and dephosphorylating a number of different proteins via the MAPK pathway. In addition, the increase in the expression of *BAX*, which is known as a pro-apoptotic gene, suggests that MAPK plays an active role in driving A549 cells to apoptosis.

The induction of apoptosis was also supported by our RT-qPCR study. *GAPDH*, *BAX,* and *BCL-2* levels of the groups to which RJ and AVE were applied alone and in combination were examined. Pro-apoptotic *BAX* levels in AVE-treated A549 and HT29 cells are significantly higher than in other groups, indicating that AVE affects apoptosis. When *BAX* and *BCL-2* levels are compared in A549 cells, a significant difference is revealed. The fact that antiapoptotic *BCL-2* is lower than pro-apoptotic *BAX* levels indicates that the treatments induced the apoptotic process. In HT29 cells, *BAX* levels were higher than *BCL-2* levels in RJ and RJ + AVE-treated groups, while there was no significant difference between the AVE-treated groups. This indicates that RJ acts pro-apoptotically for HT29 cells.

All of the genes (*TPI1*, *PGK1*, *TKT*, *ENO2*, *ENO1*, *ALDOA*) that have been linked to biochemical pathway mechanisms showed increased expression when the therapies' synergistic effects were assessed in the HT29 cell line. Increased cancer prognosis, metastatic process, and poor survival are observed in many cancer types, such as lung cancer, head and neck cancer, pancreatic cancer, and colon cancer with especially high expression of *ENO1* protein, which shows intracellular and extracellular activation depending on cellular localization [43] and it is important for maintaining the Warburg effect for cancer cells [44]. Our study's higher level of expression indicates that HT29 cells become resistant to the therapies they are given. Furthermore, glycolytic mechanism analyses show that the synergetic effect is almost ineffective in HT29, without causing any changes. It was thought that overexpression of *ENO1* may contribute to ATP production in HT29 cells.

On the other hand, in our study found that *MAT2A* gene expression decreased. Methionine adenosyltransferase (MAT) is a cellular enzyme that catalyzes the formation of S-adenosylmethionine (SAMe), the major biological methyl donor. In mammals, this essential enzyme is the product of two distinct genes, *MAT1A* and *MAT2A*, which exhibit a different expression pattern between different tissues. *MAT1A* encodes α1, which forms dimers (*MATIII*) and tetramers (*MATI*), predominantly expressed in liver parenchyma cells, while *MAT2A* encodes the α2 catalytic subunit of the *MATII* isoenzyme, which is expressed in all other tissues. Furthermore, human liver and colon cancers are reported to have higher *MAT2A* expression. Most studies, they were reported that decreasing *MAT2A* expression induces apoptosis, cell cycle arrest, and lower cell proliferation [45–47].

These analyses show that decreased expression of some genes, especially in HT29, has a negative effect on cancer cells, while genes with increased expression have a positive contribution or vice versa. Therefore, the synergetic effects of the treatments used may vary from cell to cell and according to cancer type. According to the literature, it is known that chemotherapeutic drugs used for cancer treatments create resistance in cancer cells after they are used for long time. For this reason, natural components and the combined of them has been studied recently. In some studies, it has been found that natural compounds have a significant effect on cancerous cells. It has been concluded that natural compounds increase the sensitivity of cancer cells and are more effective in combination treatments [48–50]. In our study, when the glycolytic mechanism, apoptosis, and transcriptome profile were evaluated together for the combinations of these drugs, it suggested that they had more positive effects on the therapeutic process in A549.

## Conclusion

The results of the experiments show that both Royal Jelly (RJ) and the combination with *Aloe vera* (AVE) significantly reduced the glycolytic capacity in lung cancer cells. This observation is especially important in the context of the Warburg effect, where cancer cells preferentially use glycolysis for energy production. The combination of RJ and AVE effectively reduced the high glycolytic activity characteristic of untreated lung cancer cells. In contrast, while AVE alone was found to increase the glycolytic capacity in lung cancer cells, the presence of royal jelly was seen to significantly reduce this effect. In contrast, the interaction of RJ and AVE exhibited a different outcome in colon cancer cells. AVE alone was effective in reducing the glycolytic capacity within this cell type; however, the combination treatment of RJ and AVE did not yield a similar reduction in the glycolytic pathway. Instead, it appeared to amplify the effects of RJ when administered alone. In this context, AVE was observed to lower the energy demands of colon cancer cells. Moreover, a comparative analysis revealed that the treatment with RJ and the combination of RJ and AVE resulted in decreased levels of the antiapoptotic protein BCL-2 relative to the pro-apoptotic protein BAX in lung cancer cells. This suggests that the inhibition of the glycolytic pathway and the subsequent induction of apoptosis underscore the therapeutic potential of these compounds. When assessing colon cancer cells under similar conditions, elevated BAX levels were noted in both the RJ- and RJ + AVE treatment groups, while no significant variation was observed in the AVE treatment group. The genetic and metabolic profiles of HT29 cells often make them less susceptible to single-agent therapies. By using a combination of agents, such as chemotherapeutics and natural compounds, it is possible to simultaneously target multiple pathways involved in tumor growth and survival. This approach not only synergistically improves overall antitumor activity but also reduces the likelihood of developing resistance. Therefore, the efficacy of combination therapy with natural compounds to treat cancer needs to be further investigated.

## Supplementary Information

Below is the link to the electronic supplementary material.

Supplementary file1 (XLSX 110 KB)

## Data Availability

The data is available in this study and can be obtained from the corresponding author if requested. No datasets were generated or analyzed during the current study.
